# Diagnostic and genomic characterization of an imported chikungunya virus case in Ningxia Hui Autonomous Region, China, September 2025

**DOI:** 10.1016/j.imj.2026.100242

**Published:** 2026-03-03

**Authors:** Jianxin Pei, Jingting Wang, Ling Niu, Ting Mu, Ziyang Luo, Shanshan Du, Huiqin Wang, Yuefen Zhang, Jiandong Li, Zhonglan Wu

**Affiliations:** a Ningxia Hui Autonomous Region Academy of Preventive Medicine, Ningxia Center for Disease Control and Prevention, Yinchuan 750004, China; b Clinical laboratory Department, Zhongwei Center for Disease Control and Prevention, Zhongwei 755099, China; c National Institute for Viral Disease Control and Prevention, Chinese Center for Disease Control and Prevention, Beijing 102211, China

**Keywords:** Chikungunya virus, Imported infection, *Aedes albopictus*, Whole-genome sequencing, Phylogenetic analysis, Ningxia Hui Autonomous Region

## Abstract

To analyze the first imported case of chikungunya virus (CHIKV) in Ningxia Hui Autonomous Region (Ningxia), China, and to provide insights for the prevention and control of emerging infectious diseases. An epidemiological investigation and viral detection was performed via quantitative reverse transcription polymerase chain reaction (qRT-PCR). The virus was further characterized using whole-genome sequencing and phylogenetic analysis. A 26-year-old female was confirmed to be infected with CHIKV. Genotypic analysis identified the East/Central/South African (ECSA) Indian Ocean Lineage (IOL), specifically the ECSA2 sub-lineage. A key mutation (E1-A226V), known to enhance viral adaptability to *Aedes albopictus*, was identified. Phylogenetic analysis revealed 99.21% nucleotide similarity to a CHIKV strain isolated in Foshan City, Guangdong Province, in 2025. No *Ae. aegypti* or *Ae. albopictus* were found in Ningxia. This imported case of CHIKV in Ningxia, genetically linked to the ECSA-IOL lineage carrying the E1-A226V mutation, underscores the risk of introduction and potential local transmission of arboviruses via domestic travel. Enhanced surveillance, timely diagnosis, and effective management of imported cases are essential for preventing outbreaks.

## Highlights

• First imported chikungunya case documented in Ningxia, China, September 2025.
• Patient had recent travel history to Shandong Province before symptom onset.
• Whole-genome sequencing identified ECSA Indian Ocean Lineage (ECSA2).
• Key E1-A226V mutation enhancing Ae. albopictus transmission detected.
• Strain shared 99.21% nucleotide identity with 2025 Foshan outbreak isolate.

## Graphical abstract

**Figure:**
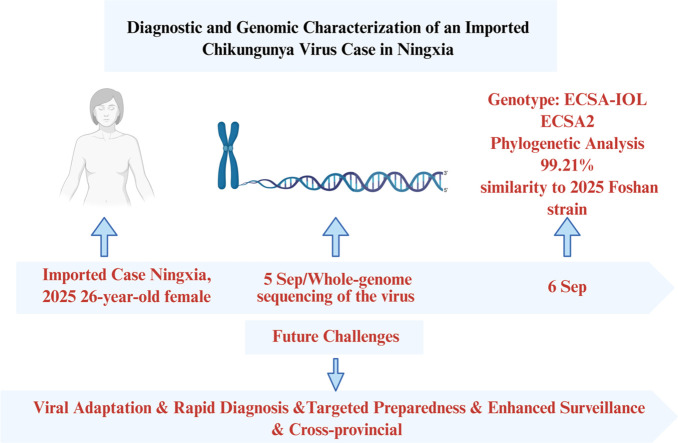

## 1 Introduction

Chikungunya virus (CHIKV) is a mosquito-borne arbovirus that causes a debilitating febrile illness often accompanied by severe arthralgia. It is primarily transmitted by *Aedes aegypti* and *Ae. albopictus* mosquitoes.^1^ Since its re-emergence in the 21st century, CHIKV has demonstrated significant potential for global expansion. In China, the virus has shifted from sporadic imported cases to localized outbreaks, with Guangdong Province being the most affected region. The initial outbreak occurred in Dongguan City in 2010.^2^ More recently, a large-scale outbreak emerged in Foshan City in 2025, infecting over 10,000 people and indicating sustained local transmission with a high basic reproduction number (R_0_).^3^^,^^4^ The ongoing Foshan outbreak has been driven by the East/Central/South African (ECSA) Indian Ocean Lineage (IOL), with genomic studies confirming the presence of adaptive mutations such as E1-A226V, which significantly enhances viral fitness in *Ae. albopictus*.^5^, ^6^, ^7^

Ningxia Hui Autonomous Region (Ningxia), located in western China, has a population of approximately 7.3 million and has never reported a case of chikungunya (CHIK) infection. The region does not routinely monitor *Ae. albopictus* mosquitoes. On September 4, 2025, an imported CHIK case was confirmed in Zhongwei City, marking the first reported case in Ningxia. Although *Ae. albopictus* has not been detected in Ningxia through the national vector surveillance system since 2018, it is established in most of China. When environmental conditions are favorable, imported cases may lead to local transmission of the virus, as demonstrated by CHIK outbreaks in China since 2010,^6^ particularly the 2025 outbreak in Foshan City.^1^ This underscores the risk of further autochthonous CHIK transmission in the Chinese mainland. Therefore, the triad of timely case detection, standardized mosquito surveillance, and risk-based epidemiological investigations forms the cornerstone of effective epidemic prevention and control.

## 2 Case presentation

### 2.1 Patient information

A 26-year-old Chinese woman visited the Zhongwei Center for Disease Control and Prevention (CDC) on September 4, 2025, for testing for the CHIKV due to symptoms of fever and joint pain. The patient and her husband had traveled to Shandong Province during the week prior to the onset of illness. They flew to Shandong Province on August 27, arriving in Qingdao City early on August 28. They then traveled to Zouping City, where she reported being bitten by a mosquito the following day. On August 30, she visited several sites in Zibo City, including Tanxi Mountain. On August 31, she flew from Jinan City to Zhongwei City, arriving on the afternoon of September 1, after which she remained at home.

### 2.2 Clinical indicators

On September 3, she developed arboviral symptoms, including low-grade fever and joint pain. The patient presented with a temperature of 38.5°C, a heart rate of 78 bpm, a respiratory rate of 18 breaths per minute, and a blood pressure of 130/80 mmHg. The clinical course progressed to include hallmark manifestations of CHIKV infection, characterized by significant polyarthralgia and a maculopapular rash on the lower extremities. Laboratory evaluation demonstrated a normal white blood cell count (5.30 × 10^9^/L) with neutrophilic predominance (73.1%) and a preserved platelet count (224,000/L), effectively excluding dengue hemorrhagic fever. Biochemical profiling showed an isolated mild elevation of aspartate aminotransferase (AST, 42 IU/L), suggesting subclinical hepatic involvement, while alanine aminotransferase (ALT), renal function parameters, and inflammatory markers (erythrocyte sedimentation rate [ESR], 3 mm/hr; C-reactive protein [CRP], 6.74 mg/L) remained within normal limits.

## 3 Results

### 3.1 Confirmation of CHIK infection and epidemiological investigation

Upon the patient's presentation at the Zhongwei CDC, blood samples were collected. Epidemiological information was obtained from her and her husband, covering the period from August 24 to September 4. The Ningxia CDC conducted laboratory testing using quantitative reverse transcription polymerase chain reaction for differential diagnosis, which included screening for dengue virus (DENV), Dabie bandavirus (DBV) and CHIKV. The patient tested positive for CHIKV, while tests for DENV and DBV were negative. Following this confirmation—marking the first documented CHIKV case in the region—she was hospitalized for isolation to prevent mosquito exposure. Concurrently, a local screening campaign and enhanced mosquito surveillance were initiated. Monitoring results indicated that no *Ae. aegypti* or *Ae. albopictus* mosquitoes were detected in the local area.

The patient received supportive care during a three-day hospitalization, including acetaminophen for analgesia and antipyresis, maintained adequate hydration, and underwent continuous clinical monitoring. She was discharged on September 12 in stable condition following the resolution of the febrile illness, with persistent arthralgia requiring outpatient follow-up to monitor joint symptom resolution and transaminase levels.

### 3.2 Whole-genome sequencing and genomic characterization

**Fig. 1:**
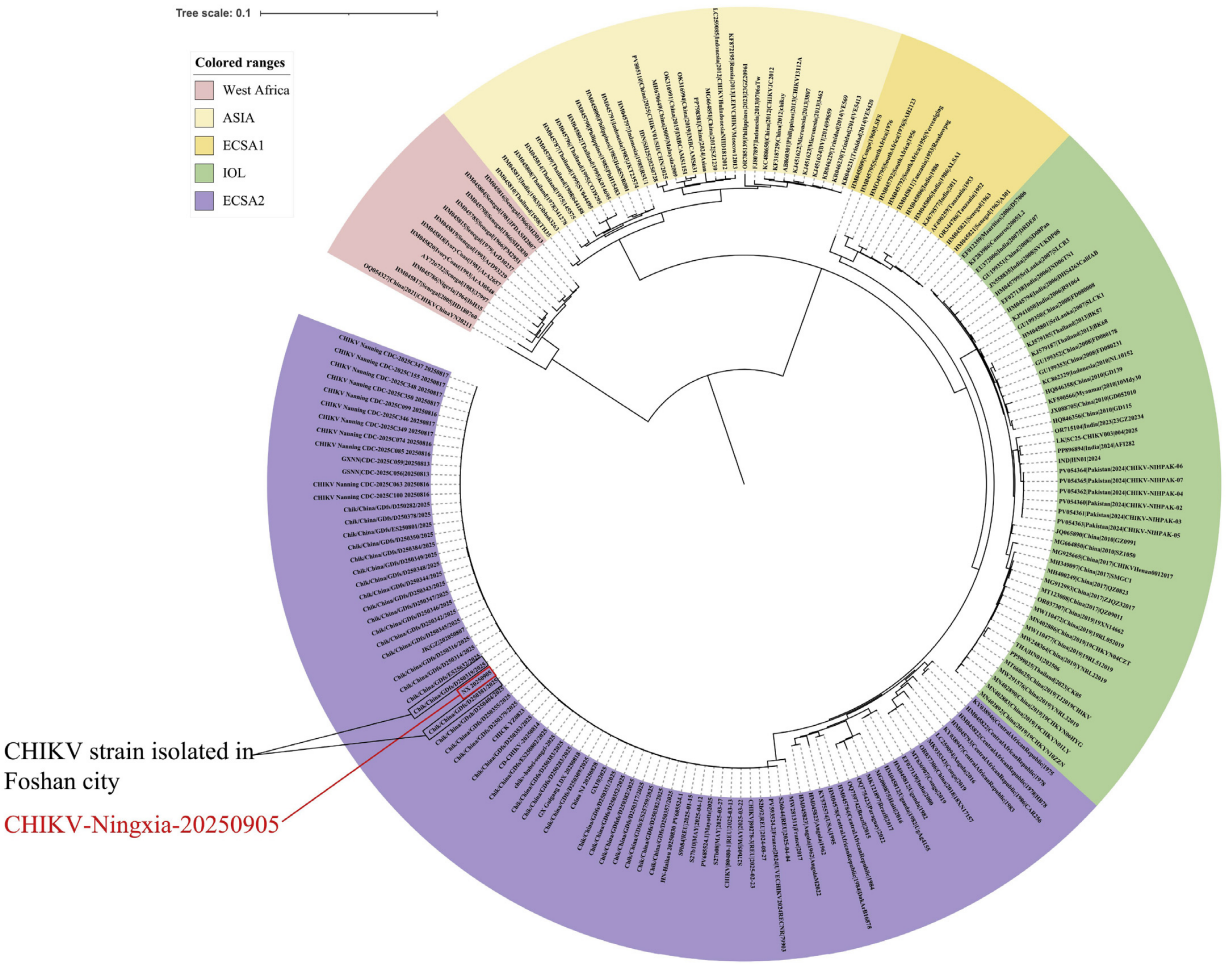
Phylogenetic tree based on the complete genome sequence of the CHIKV strain from Ningxia and related global strains. *Note*: The branch of the imported Ningxia case is indicated by a red line, and the closely related strain from Foshan City, Guangdong Province, is indicated by a black dashed line. *Abbreviations*: ASIA, Asian lineage; ECSA1, East/Central/South African lineage 1; IOL, Indian Ocean Lineage; ECSA2, East/Central/South African lineage 1; CHIKV, chikungunya virus.

Whole-genome sequencing of CHIKV was performed using a CHIKV ultra-sensitive target capture kit (Beijing Weifuture Technology Co., Ltd. Beijing, China, No: B-17033) on a next-generation sequencing platform. Sequencing commenced at 20:00 on September 4 and was completed by 18:00 on September 5, yielding a complete genome sequence of 11,805 bp. Genotypic analysis identified the virus as belonging to the ECSA IOL, specifically the ECSA2 sub-lineage. A notable nonsynonymous mutation in the E1 glycoprotein gene, E1-A226V, which is associated with enhanced viral adaptability to and transmission by *Ae. albopictus*, was observed. Phylogenetic analysis based on the complete genome sequence revealed that the Ningxia strain shared 99.21% nucleotide sequence identity with a CHIKV strain isolated in Foshan City, Guangdong Province, in 2025. The sequence has been submitted to the National Genomics Data Center (Accession No.: C_AA130858.1) (Fig. 1).

## 4 Discussion

This first imported case of CHIKV in Ningxia underscores the ongoing risk of arboviral introduction into non-endemic regions of China via domestic travel. The genomic identification of an ECSA-IOL strain carrying the E1-A226V mutation—a key adaptation linked to the large 2025 outbreak in Foshan City—confirms that imported viruses can possess the inherent capacity for efficient transmission by *Ae. Albopictus*.^5^^,^^7^ Although no established Aedes populations were detected in Ningxia during this investigation, the presence of a lineage with enhanced vector adaptability highlights the importance of proactive genomic and entomological surveillance in regions currently considered low-risk.

The patient's rapid movement from an area of active transmission (Shandong Province) to a non-endemic region (Ningxia) exemplifies a critical pathway for viral spread. Implementing a robust cross-provincial travel health alert system, while facing practical challenges in coordination and resource allocation, could be initiated through a phased, risk-prioritized approach. Focusing on high-risk travel corridors and seasonal outbreak patterns would facilitate early warnings to destination regions, enabling preemptive screening, targeted vector control, and public health preparedness.

Climate change is a potential influencing factors of autochthonous transmission risk. Projected warming trends are anticipated to shift the suitable habitat range of *Ae. albopictus* northwards, potentially extending into regions such as Ningxia in the coming decades.^8^ This ecological shift necessitates a forward-looking strategy, including the early establishment of sentinel mosquito monitoring sites in potentially at-risk areas before vector invasion occurs. Integrating seasonal climate forecasts with public health planning can help identify high-risk periods and locations, allowing for the timely deployment of interventions.

Furthermore, strengthening rapid diagnostic capacity at primary healthcare facilities, particularly in gateway areas and non-endemic regions, is essential for the timely differentiation of CHIKV from other febrile illnesses like dengue.^9^ Equipping frontline clinics with CHIKV rapid tests can significantly reduce the time to diagnosis, enabling immediate isolation and mosquito-avoidance measures to prevent the initiation of local transmission chains.

This case illustrates the convergence of travel-mediated virus introduction and climate-influenced vector range expansion as dual drivers of arboviral transmission risk in China. A comprehensive preparedness strategy, integrating vigilant surveillance (syndromic, pathogen, and vector), travel-based alert systems, rapid frontline diagnostics, and proactive vector monitoring in climatically susceptible regions, is essential to prevent the establishment and spread of CHIK and other mosquito-borne diseases.

Limitations: Although molecular tracing identified the ECSA-IOL lineage, the retrospective epidemiological investigation could not determine the precise timing, location or events that led to the patient's infection, which could introduce potential recall bias.

## 5 Conclusions

This first imported case of CHIK in Ningxia highlights the increasing risk of local transmission of mosquito-borne diseases in China, driven by the convergence of domestic travel and climate change. The identification of an ECSA-IOL strain with the A226V mutation, highly homologous to the 2025 Foshan outbreak strain, confirms that imported viruses have the potential for efficient transmission by *Ae. albopictus*. Strengthening core pillars of preparedness—vigilant surveillance, travel-based alerts, rapid frontline response, and proactive vector monitoring in climatically vulnerable areas—is essential to mitigate the threat of CHIK and other arboviral diseases in China.

## CRediT authorship contribution statement

**Jianxin Pei:** Writing – original draft, Data curation, Conceptualization. **Jingting Wang:** Resources, Investigation, Data curation. **Ling Niu:** Data curation. **Ting Mu:** Data curation, Conceptualization. **Ziyang Luo:** Data curation, Conceptualization. **Shanshan Du:** Software. **Huiqin Wang:** Data curation. **Yuefen Zhang:** Data curation. **Jiandong Li:** Writing – review & editing, Software, Resources, Conceptualization. **Zhonglan Wu:** Writing – review & editing, Writing – original draft, Conceptualization.

## Informed consent

Informed consent was obtained from the patient to publish any accompanying data and images.

## Organ donation

Not applicable.

## Ethical statement

This study was approved by the Ethics Review Committee of the Ningxia Center for Disease Control and Prevention (No. 2025-LLSC-227).

## Data availability statement

All data and materials generated in this study are retained by the first and corresponding authors and are available upon reasonable request. The complete genome sequence has been deposited in the National Genomics Data Center under accession number C_AA130858.1.

## Animal treatment

Not applicable.

## Generative AI

Not applicable**.**

## Funding

This study was supported by the 2024 National Public Health Leading Talents Program (No. 2024005) and the Ningxia Natural Science Foundation (No. 2024AAC03734).

## Declaration of competing interest

The authors declare that they have no competing interests.
